# Growth differentiation factor 15: a valuable biomarker for the diagnosis and prognosis of late-onset form of multiple Acyl-CoA dehydrogenation deficiency

**DOI:** 10.1186/s13023-025-03651-1

**Published:** 2025-04-03

**Authors:** Sun Yuan, Tang Shuyao, Lyu Jingwei, Wen Bing, Xu Jingwen, Li Busu, Zhao Bing, Ji Kunqian, Yan Chuanzhu

**Affiliations:** 1 Neurology, Cheeloo College of Medicine, Qilu Hospital, Shandong University, Qingdao, 266035 China; 2 Department of Neurology, Shandong Key Laboratory of Mitochondrial Medicine and Rare Diseases, Research Institute of Neuromuscular and Neurodegenerative Diseases, Cheeloo College of Medicine, Qilu Hospital, Shandong University, Jinan, 250012 Shandong China; 3 Shandong Key Laboratory: Magnetic Field-free Medicine & Functional Imaging, Shandong University, Jinan, 250012 Shandong China

**Keywords:** Multiple Acyl-CoA dehydrogenation deficiency, Growth differentiation factor 15, Mitochondrial dysfunction, Integrated stress response, Biomarker

## Abstract

**Background:**

Multiple acyl-CoA Dehydrogenation Deficiency (MADD) is a hereditary metabolic disorder affecting the metabolism of fatty acids, amino acids, and choline, typically presenting with fat accumulation and mitochondrial abnormalities in muscle pathology. Growth differentiation factor 15 (GDF15) is a stress-responsive cytokine implicated in energy metabolism. Therefore, this study aimed to assess the level of GDF15 in patients with late-onset MADD and to evaluate its potential as a reliable biomarker for diagnosing symptoms and determining the severity of late-onset MADD.

**Methods:**

In this study, consecutive patients with MADD mitochondrial diseases were recruited from the Neuromuscular Center of Qilu Hospital, Shandong University, between April 2015 and October 2021. We measured serum GDF15 levels in patients with late-onset MADD and healthy controls. Additionally, we analyzed the messenger RNA(mRNA) expression of GDF15 and integrated stress response (ISR)-related factors, including CHOP, ATF5, and TRIB3, in the muscles.

**Results:**

Serum GDF15 levels in patients with late-onset MADD were 18.8 times higher than those in healthy controls. GDF15 levels decreased as the disease progressed, and its elecated levels correlated with anorexia symptoms. The mRNA expression of GDF15 and ISR-related factors in the muscles was higher in patients with late-onset MADD than in controls.

**Conclusion:**

GDF15 levels were significantly elevated in symptomatic patients with late-onset MADD, likely due to mitochondrial dysfunction activating the ISR pathway. These findings suggest that GDF15 is a valuable biomarker for monitoring disease severity and symptomatology in patients with late-onset MADD.

**Supplementary Information:**

The online version contains supplementary material available at 10.1186/s13023-025-03651-1.

## Background

Multiple acyl-CoA dehydrogenation deficiency (MADD) is an autosomal recessive genetic disorder characterized by disruptions in the metabolism of fatty acids, amino acids, and choline [1]. Clinically, MADD is classified into three types: type I (neonatal onset with congenital anomalies), type II (neonatal onset without congenital anomalies), and type III (late onset) [2]. Late-onset MADD is heterogeneous in both genetic and phenotypic manifestations. Common symptoms include muscle weakness, exercise intolerance, hepatomegaly, vomiting episodes, hypoglycemia, and metabolic acidosis, along with occasional rhabdomyolysis or severe sensory neuropathy in some cases.

The diagnosis of MADD is typically indicated by elevated levels of several acylcarnitine species in the blood and increased excretion of multiple organic acids in the urine, confirmed through the identification of biallelic pathogenic variants in the genes encoding electron transfer flavoprotein α(ETFA), electron transfer flavoprotein β(ETFB), or electron transfer flavoprotein dehydrogenase (ETFDH) [2]. Many cases of late-onset MADD, known as riboflavin-responsive MADD (RR-MADD) [3], are associated with mutations in ETFDH, which account for approximately 80% of RR-MADD cases, particularly prevalent in the Chinese population. The clinical phenotype and severity of late-onset MADD varies widely, ranging from mild weakness to severe quadriplegia and respiratory failure, with symptoms manifesting from infancy to adulthood.

Previous studies have suggested that RR-MADD development is influenced by both ETFDH mutations and disruptions in flavin adenine dinucleotide(FAD) homeostasis [3]. Except for the characteristic feature of fat accumulation, mitochondrial abnormalities, such as ragged red fibers (RRFs) and succinate dehydrogenase (SDH)-deficient fibers [4], are commonly observed in the muscle pathology of late-onset MADD. Additionally, mitochondrial dysfunction and oxidative stress dysregulation are believed to play key roles in the pathogenesis [1].

Growth differentiation factor 15 (GDF15) is a stress-responsive cytokine within the transforming growth factor beta (TGF-β) superfamily, playing roles in inflammation, metabolism, and cancer [5]. It is recognized as a key regulator of energy metabolism and is upregulated in response to mitochondrial dysfunction [6]. Additionally, GDF15 binds to the glial cell line-derived neurotrophic factor receptor alpha-like (GFRAL) receptor, which interacts with its tyrosine kinase coreceptor rearranged during transfection (RET) to initiate a signaling cascade that may activate various intracellular pathways [7]. The GDF15–GFRAL axis has been shown to regulate food intake, confer resistance to obesity, promote lipolysis in adipose tissue, and enhance insulin sensitivity [7–9]. Previous studies have suggested that elements of the cellular integrated stress response (ISR) are involved in regulating GDF15 expression [10], while the ISR can be triggered by mitochondrial dysfunction and oxidative stress.

However, no study has specifically examined GDF15 levels in late-onset MADD to date. Therefore, this study aimed to determine the concentration of GDF15 levels in late-onset MADD and assess its potential as a biomarker for evaluating disease symptoms and severity.

## Methods

### Participants

Consecutive patients with MADD and mitochondrial diseases (MDs) were recruited from the Neuromuscular Center of Qilu Hospital, Shandong University, between April 2015 and October 2021. Patients with MADD were diagnosed via muscle biopsy and confirmed to have ETFDH mutations, whereas those with MDs were diagnosed based on standard criteria for MD. Healthy controls were confirmed to be free of muscle-related diseases following comprehensive testing at our center. We collected clinical presentation and gene mutations of the patients with late-onset MADD, as shown in Table 1.

**Table 1:** Clinical presentation and gene mutations of the patients with MADD

Case	sex	onset age	CK(U/L)	LDH (U/L)	Hcy (mmol/L)	EMG	anorexia	ETFDH mutations	
1	M	29	400–500	UA	UA	NL	Yes	c.770A > G p.Y257C	c.1157G > T p.G386V
2	F	31	81	436	UA	NL	No	c.389A > T p.D130V	c.1395T > G p.Y465stop
3	F	18	2909	UA	UA	NL	No	c.152G > A p.R51Q	c.152G > A p.R51Q
4	M	22	259	UA	UA	myopathic	Yes	c.1227A > C p.L409F	c.1227A > C p.L409F
5	F	20	7856	3621	99.7	NL	Yes	c.250G > A p.A84T	c.389A > T p.D130V
6	F	51	342	UA	UA	UA	Yes	c.1205C > T p.T402I	c.1411A > G p.T471A
7	M	17	3320	UA	UA	peripheral neuropathy	No	c.770A > G p.Y257C	c.872T > G p.V291G
8	F	19	1998	UA	UA	NL	Yes	c.1211T > Cp.M404T	Transcript↓
9	M	24	268	370	26	peripheral neuropathy	No	c.829_830insG p.E277G stop*6	duplication in exon4,5
10	M	73	513	UA	UA	abnormal H-reflex	No	c.1227A > C p.L409F	c.1084G > A p.G362R
11	M	33	2056	1319	UA	abnormal H-reflex	Yes	c.250G > A p.A84T	c.250G > A p.A84T
12	F	20	2823	2046	24.9	myopathic	No	c.1205C > T p.T402I	c.805C > G p.Q269E
13	M	65	1486.6	1023	UA	myopathic	No	c.1227A > Cp.L409F	c.1211T > Cp.M404T
14	M	14	928	1081	39.1	UA	Yes	c.857G>T p.W286L	c.1669G>Ap.E557K
15	F	16	1267	3460	UA	UA	No	c.1395dupT p.G466Wstop*24	c.998A > G p.Y333C
16	M	14	35/803	UA	UA	UA	No	c.389A > T p.D130V	c.920C > G p.S307C
17	F	41	772	811	UA	UA	Yes	c.250G > A p.A84T	c.1227A > C p.L409F
18	F	65	102	302	50	myopathic	No	c.770A > G p.Y257C	c.1227A > C p.L409F
19	M	12	194	259	18.6	NL	Yes	c.1227A > C p.L409F	c.G353T p.C118F
20	M	27	2553	UA	UA	UA	Yes	c.250G > A p.A84T	c.A389T p.D130V
21	M	19	243	390	15.61	myopathic	No	c.1531G > A p.D511N	c.1395T > G p.Y465stop
22	M	22	622	442	27.66	UA	No	c.250G > A p.A84T	c.250G > A p.A84T
23	M	40	252	766	UA	myopathic	No	c.770A > G p.Y257C	ND
24	M	13	1371	692	UA	myopathic	Yes	c.250G > A p.A84T	c.487G>A p.G163R
25	M	29	99	UA	UA	NL	Yes	c.1411A > G p.T471A	c.1448C > T p.P483L
26	M	20	124	374	UA	UA	Yes	c.1454C>G p.T485S	c.242T>C p.L81P
27	M	47	1039	892	16.26	myopathic	Yes	c.770A > G p.Y257C	c.872T > G p.V291G
28	F	48	588	2042	8.3	myopathic	No	c.250G > A p.A84T	c.1017G > C p.E339D
29	F	17	12841	5979	22.8	NL	Yes	c.872T>G p.V291G	c.770A > G p.Y257C
30	M	24	1964	2655.6	UA	myopathic	Yes	c.910T>A p.T304A	c.910T>A p.T304A
31	M	36	433	408	29.1	myopathic	No	c.770A > G p.Y257C	c.872T > G p.V291G
32	M	14	339	552	9.84	myopathic	No	c.1395T>G p.Y465*	c.1227A > C p.L409F
33	M	30	316	525	43.63	myopathic	No	c.1327T>C p.W443R	c.1399G > C p.G467R
34	M	20	236	312	44.2	myopathic	No	c.389A > T p.D130V	c.251C > T p.A84V
35	F	66	8568	6632	2.53	myopathic	Yes	c.1295T > A p.V432E	c.1531G > A p.D511N
36	F	66	1188	1441	UA	myopathic	Yes	c.3G>C p.0?	c.1454C>G p.T485S
37	F	17	362	408	11.8	UA	No	c.770A > G p.Y257C	c.251C > T p.A84V
38	F	15	70	878	10.6	UA	Yes	c.92C>T p.Thr31Ile	c.92C>T p.Thr31Ile
39	M	54	759	394	37.7	neuropathic	No	c.1395T>G p.Y465*	c.335C>G p.L119V
40	F	32	433	857	16.4	UA	No	c.A389T p.D130V	c.250G > A p.A84T
41	M	45	197	3065	UA	myopathic	Yes	c.1211T > Cp.M404T	c.171 G>A p.Trp57*

Note: M: male; F: female; UA: unavailable; NL: normal; ND: not detected; CK: creatine kinase; LDH: lactate dehydrogenase; HCY: homocysteine

### Procedures

#### Serum GDF15 quantification by enzyme-linked immunosorbent assay (ELISA)

Blood samples from patients were centrifuged at 3000 rpm for 10 min, and the serum was immediately frozen at − 80 °C until analysis. Serum GDF15 levels were measured using a Human GDF-15 Quantikine ELISA Kit (Catalog #: DGD150, R&D Systems) according to the manufacturer’s protocol.

#### Western blot analysis

Western blot analysis was conducted on proteins extracted from muscle homogenates of patients using a monoclonal mouse anti-GDF15 antibody (G-5) (sc-377195, Santa Cruz Biotechnology), polyclonal rabbit anti- electron transfer flavoprotein-ubiquinone oxidoreductase (ETF-QO) antibody (11109-1-AP, Proteintech Group), and monoclonal mouse anti-β-actin antibody (66009-1-Ig, Proteintech Group). After incubation with the primary antibody, horseradish peroxidase-conjugated secondary antibodies were added. Protein bands were visualized using enhanced chemiluminescence and quantified using ImageJ software. The procedures for protein extraction, protein concentration measurements, and western blot analysis are well-documented in the literature.

#### Real-time quantitative PCR

After RNA extraction from the muscle homogenates of the patients, the concentration of total RNA was measured by spectrophotometry and reverse-transcribed using an RT-PCR kit (R323; Vazyme). Real-time quantitative PCR was conducted on ABI Prism 7900HT (Applied Biosystems) with SYBR^®^ Green (Q711, Vazyme) as the fluorescent dye. The primers used for qRT-PCR are shown in the Supplementary Table 1. Amplification conditions were as follows: 95 °C for 30 s, followed by 40 cycles of 95 °C for 10 s and 60 °C for 30 s. Relative transcript abundance was analyzed using the 2^−ΔΔCt^ method, with data normalized to glyceraldehyde-3-phosphate dehydrogenase (GAPDH). Melting curve analysis was performed after each experiment.

### Statistical analysis

Data are presented as median values. Statistical analyses were performed using SPSS software. A two-tailed unpaired Student’s t-test was used for between-group comparisons, while a one-way analysis of variance (ANOVA) followed by Tukey’s post-hoc test was used for comparisons involving three or more groups. Statistical significance was set at *P* < 0.05. with significance levels indicated as follows: (*P < 0.05; ** *P* < 0.01; *** *P* < 0.001; **** *P* < 0.0001).

## Results

### Serum GDF15 concentrations are significantly elevated in late-onset MADD patients

A total of 110 serum samples were analyzed, including 41 from patients with MADD, 46 from healthy controls, and 23 from patients with MD. Demographics of three study groups were shown in supplementary Tables 2 and clinicopathological characteristics of patients with MDs were shown in supplementary Table 3. Among the 41 patients with MADD, 33 samples were collected before the administration of Vitamin B2(VitB2), and 18 samples were obtained after more than 2 months of VitB2 supplementation. Pre- and post-treatment samples were obtained for 10 patients whose clinical detail variations of patients before and after treatment were shown in supplementary Table 4.

**Fig. 1:**
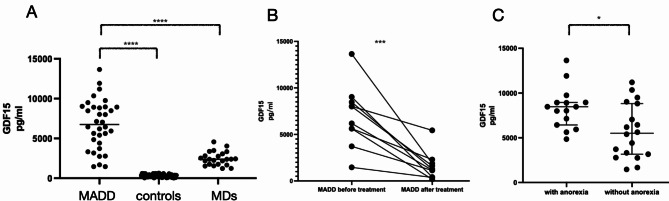
Serum GDF15 concentrations in different groups. (**A**) Serum GDF15 concentrations in healthy controls, patients with MDs, and patients with MADD; (**B**) Serum GDF15 concentrations in 10 patients with MADD before treatment and after two months VitB2 treatment; (**C**) Serum GDF15 concentrations in MADD patients with or without anorexia or decreased appetite

The median serum GDF15 concentration in untreated patients with MADD was 7045 pg/mL, significantly higher than that in patients with MDs (2384 pg/mL), and healthy controls (374 pg/mL) (Fig. 1A). After 2 months of VitB2 treatment, the MADD symptoms completely resolved, and serum GDF15 levels showed a marked decrease from 7094pg/mL pre-treatment and 1219 pg/mL post-treatment (Fig. 1B). Patients with MADD with anorexia had significantly higher GDF15 levels (8412pg/mL) than those without anorexia (5513 pg/mL) (Fig. 1C). Demographics of patients with or without anorexia were shown in supplementary Table 5.

### mRNA expression of GDF15 and ISR related factors in muscle tissue are elevated in patients with MADD

**Fig. 2:**
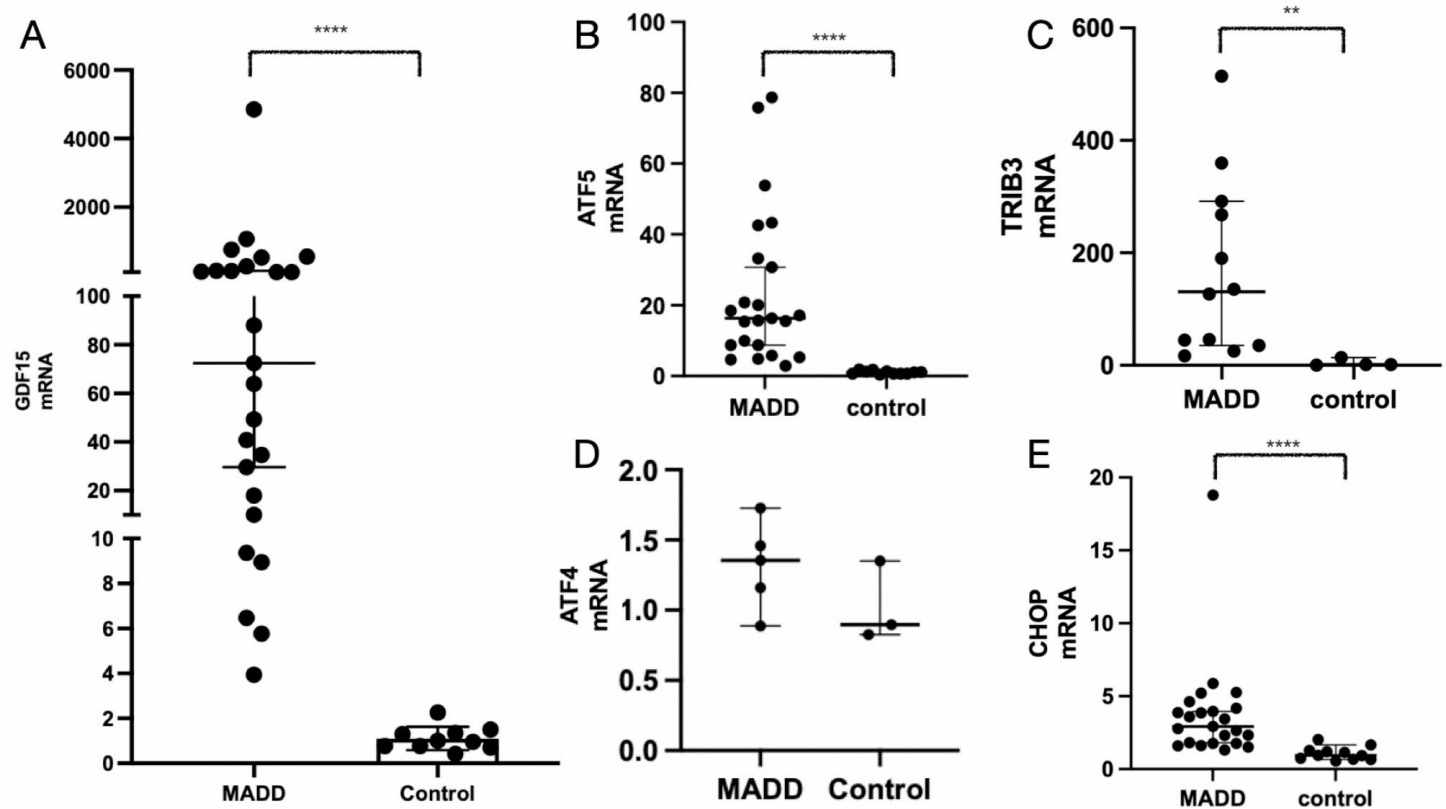
mRNA expression of *GDF15* and ISR-related genes. (A) mRNA expression of *GDF15* in patients with MADD and controls; (B) mRNA expression of *ATF5* in patients with MADD and controls; (C) mRNA expression of *TRIB3* in patients with MADD and controls; (D) mRNA expression of *ATF4* in patients with MADD and controls; (E) mRNA expression of *CHOP* in patients with MADD and controls. *P < 0.05; ** *P* < 0.01; *** *P* < 0.001; **** *P* < 0.0001

The mRNA expression of *GDF15* in frozen muscle tissue was significantly higher in patients with MADD than in healthy controls (Fig. 2A). Additionally, the mRNA levels of *ATF5*, *CHOP* and *TRIB3*, which are genes believed to be associated with *GDF15* expression were markedly elevated in the muscle tissues of patients with MADD compared to healthy controls. *ATF4* mRNA expression showed no significant difference between patients with MADD and healthy controls (Fig. 2B-E).

### Expression of GDF15 protein in patients with MADD is similar to that in the healthy controls

**Fig. 3:**
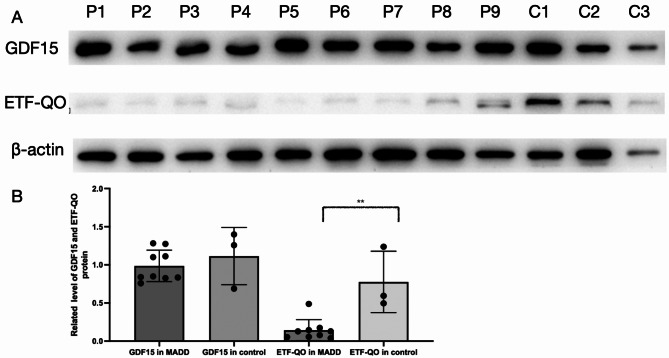
Western blot of GDF15 and ETF-QO in muscles. (**A**) Western blot analysis of GDF15 and ETF-QO protein levels in MADD patients and controls; (**B**) Quantification of GDF15 and ETF-QO were described. C1-3, control; P1-P9, patient. ** *P* < 0.01 Additional materials

GDF15 protein levels in muscle tissues from patients with MADD were comparable to those in healthy controls. However, ETF-QO protein levels were significantly lower in patients with MADD than in healthy controls (Fig. 3A and B).

## Discussion

MADD is caused by variations in the genes encoding the electron transfer flavoprotein (ETF) or electron transfer flavoprotein-ubiquinone oxidoreductase (ETF-QO), both of which are essential for mitochondrial function [11]. A hallmark of MADD is the consistent increase in reactive oxygen species (ROS) [12], which leads to oxidative stress and activates compensatory mitochondrial quality control mechanisms to preserve mitochondrial function and maintain cellular viability [13, 14]. GDF15, a key regulator of systemic mitohormetic adaptation, plays a crucial role in energy redistribution during oxidative phosphorylation (OXPHOS) dysfunction [15]. Increased levels of GDF15 in mitochondrial diseases have been confirmed in the previous studies [16–19].

Consistent with these findings, our study revealed a significant increase in serum GDF15 levels in patients with late-onset MADD, with levels 18.8 times higher than those in healthy controls. Following VitB2 supplementation, a marked reduction in serum GDF15 levels was observed, accompanied by clinical symptoms improvement. Interestingly, GDF15 levels in patients with late-onset MADD were three times higher than those observed in mitochondrial diseases (MDs). This discrepancy may be attributed to the lipolytic effect of GDF15, a known mechanism underlying its role in metabolic regulation [15]. Since MADD is characterized by dysfunction in fatty acid metabolism, this lipolysis-promoting effect may inadvertently exacerbate fatty acid accumulation within tissues, potentially worsening the condition. Consequently, we hypothesized that the metabolic role of the GDF15–GFRAL pathway may have adverse effects on MADD.

Anorexia is a common clinical manifestation in patients with MADD, and GDF15 has been implicated in appetite suppression. Our comparative analysis demonstrated significantly higher GDF15 levels in patients with MADD with anorexia than in those without anorexia, suggesting that elevated GDF15 may contribute to the anorexia observed in these patients.

GDF15 functions as an endocrine signal synthesized by various cell types in response to the activation of ISR or other signaling pathways [10]. We hypothesized that the pronounced mitochondrial dysfunction in MADD triggers ISR activation, leading to elevated *GDF15* expression. Specifically, *GDF15* mRNA expression has been shown to be downstream of the ISR pathway, with molecules such as *ATF4* and *CHOP* frequently implicated [10, 15, 20]. In our study, we observed elevated mRNA levels of ISR-related factors, including *ATF5*, *CHOP*, and *TRIB3*, in the muscle tissues of patients with MADD, while *ATF4* expression remained unchanged. This discrepancy with previous studies [10, 21] could be explained by the potential inhibition of *ATF4* expression due to elevated *TRIB3* levels [22]; however, further research is required to elucidate the underlying mechanisms.

Despite the clear elevation in serum GDF15 levels and *GDF15* mRNA expression in the muscle tissue of patients with MADD, no significant difference in GDF15 protein levels in muscle tissue was observed between patients with MADD and healthy controls. This finding aligns with the established role of GDF15 as a secreted protein, whose primary function involves interacting with GFRAL in the brainstem’s area postrema (AP) and the nucleus of the solitary tract (NTS) to regulate metabolic processes [23–26]. The majority of the GDF15 protein is secreted extracellularly, which likely explains the lower intracellular protein concentration compared to GDF15 mRNA expression levels.

GDF15 has been shown to be elevated to varying degrees during different physiological and pathophysiological conditions [27]. Previous literature has reported GDF-15 as a biomarker in cardiovascular disease, kidney disease, liver disease, metabolic syndrome, diabetes mellitus, and sepsis [5]. Based on the above results, while elevated GDF15 in patients with MADD can be distinguished from healthy controls, it does not have a high diagnostic specificity. However, when analyzed in a specific disease context, elevated GDF15 in late-onset MADD patients with a relatively clean clinical background can serve as a powerful indicator to assist in the disease diagnosis, and be used as a monitoring indicator in follow-up assessment to evaluate disease recovery. For patients with confirmed late-onset MADD, monitoring changes in GDF15 levels is also meaningful to predicting disease recurrence.

This study has some limitations. Firstly, the available patient tissues were limited to muscle and serum, making it challenging to determine whether serum GDF15 primarily originates from muscle or other tissues. Given that the muscle is a highly energy-demanding organ and that muscle weakness is a predominant symptom in late-onset MADD, it is plausible that mitochondrial dysfunction in the muscle plays a central role in triggering ISR and upregulating GDF15 expression. Therefore, further studies using appropriate animal models could help clarify this issue. Lastly, the precise effects of GDF15 on MADD remain unclear, and further investigation is required to elucidate its exact mechanisms.

## Conclusions

Our study provides novel evidence of significantly elevated of GDF15 levels in patients with late-onset MADD, suggesting that GDF15 serve as a valuable biomarker for assessing disease progression and severity. This elevation is likely associated with the activation of ISR, driven by mitochondrial dysfunction in MADD. Future studies are warranted to further investigate the role of GDF15 and its metabolic implications in MADD.

## Electronic supplementary material

Below is the link to the electronic supplementary material.

Supplementary Material 1: Description of data: Supplementary **Fig.1**. Western blot of GDF15 in muscles. We switched to a monoclonal rabbit anti-GDF15 antibody (ZRB2590, Sigma-Aldrich) and performed Western blot again

Supplementary Material 2: Description of data: Supplementary **Table 1**. The primers used for qRT-PCR. Supplementary **Table 2**. Demographics of three study groups. Supplementary **Table 3**. Clinicopathological characteristics of patients with MDs. **Supplementary Table 4**. Clinical detail variations of patients before and after treatment. Supplementary **Table 5**. Demographics of patients with or without anorexia

## Data Availability

The datasets used and/or analysed during the current study are available from the corresponding author on reasonable request.

## Abbreviations

MADD: Multiple Acyl-CoA Dehydrogenation Deficiency
RR-MADD: riboflavin-responsive MADD
GDF15: Growth differentiation factor 15
ISR: Integrated Stress Response
ETFA: electron transfer flavoproteinα
ETFB: electron transfer flavoproteinβ
ETFDH: electron transfer flavoprotein dehydrogenase
RRFs: ragged red fibers
SDH: succinate dehydrogenase
TGF-β: transforming growth factor beta
GFRAL: glial cell line-derived neurotrophic factor receptor alpha-like
RET: Rearranged during Transfection
MDs: mitochondrial diseases
ELISA: Enzyme-linked immunosorbent assay
ETF-QO: Electron transfer flavoprotein: ubiqionone oxidoreductase
GAPDH: Glyceraldehyde-3-phosphate dehydrogenase
ANOVA: Analysis of Variance
ATF5: Activating transcription factor 5
CHOP: C/EBP homologous protein
TRIB3: Tribbles Pseudokinase 3
ATF4: Activating transcription factor 4
OXPHOS: oxidative phosphorylation
AP: area postrema
NTS: nucleus of the solitary tract
CK: creatine kinase
LDH: lactate dehydrogenase
HCY: homocysteine
ROS: reactive oxygen species
