# Supplementary figures and images for: Growth differentiation factor 15: a valuable biomarker for the diagnosis and prognosis of late-onset form of multiple Acyl-CoA dehydrogenation deficiency
Source: Orphanet J Rare Dis. 2025 Apr 3;20:159. doi: 10.1186/s13023-025-03651-1 (PMC11969926; doi:10.1186/s13023-025-03651-1)

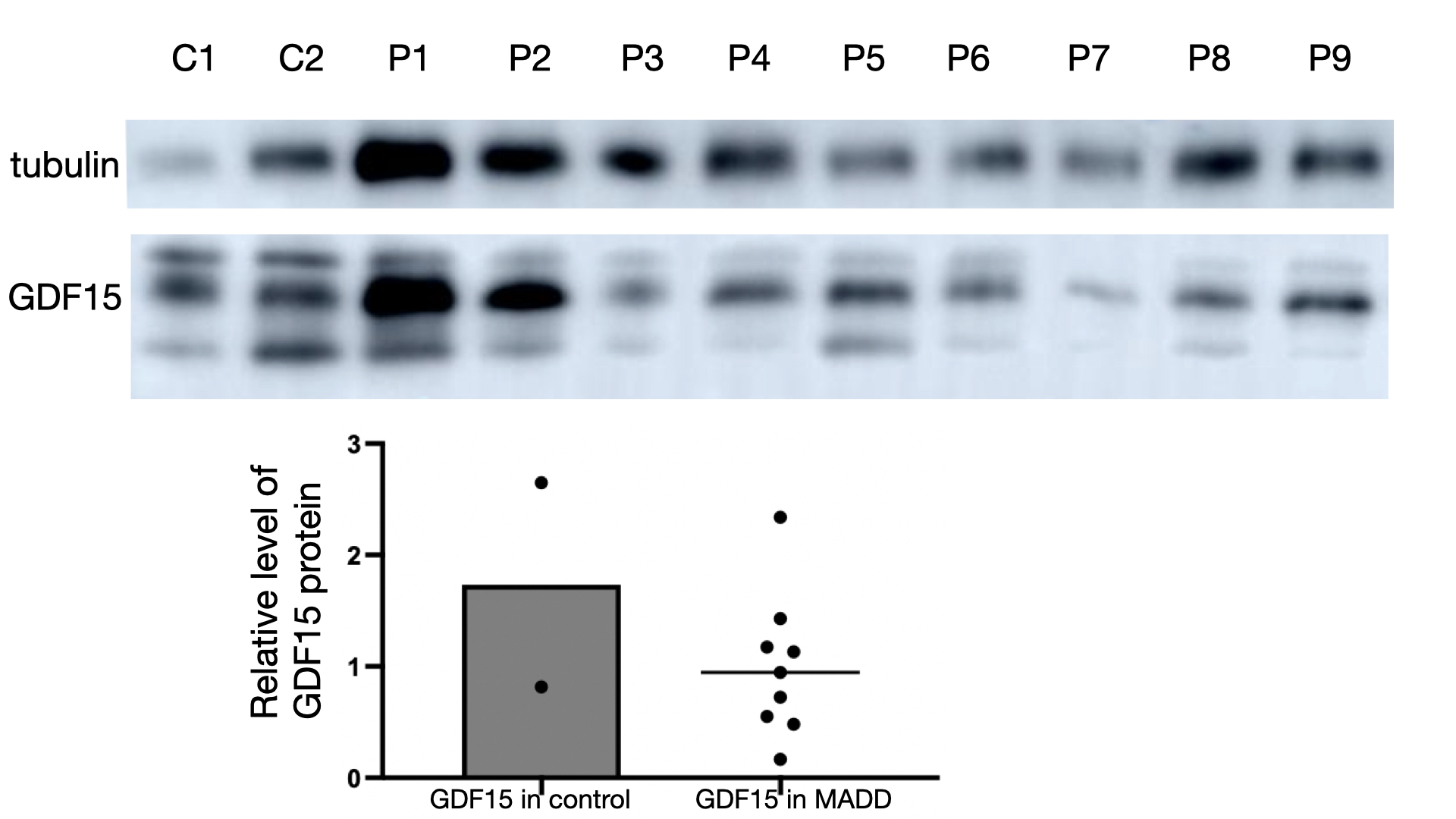

Supplement: Supplementary file 1 — Supplementary Material 1: Description of data: Supplementary Fig.1. Western blot of GDF15 in muscles. We switched to a monoclonal rabbit anti-GDF15 antibody (ZRB2590, Sigma-Aldrich) and performed Western blot again [file 13023_2025_3651_MOESM1_ESM.jpeg]
