# Supplementary material for: Growth differentiation factor 15: a valuable biomarker for the diagnosis and prognosis of late-onset form of multiple Acyl-CoA dehydrogenation deficiency
Source: Orphanet J Rare Dis. 2025 Apr 3;20:159. doi: 10.1186/s13023-025-03651-1 (PMC11969926; doi:10.1186/s13023-025-03651-1)
Supplement: Supplementary file 2 — Supplementary Material 2: Description of data: Supplementary Table 1. The primers used for qRT-PCR. Supplementary Table 2. Demographics of three study groups. Supplementary Table 3. Clinicopathological characteristics of patients with MDs. Supplementary Table 4. Clinical detail variations of patients before and after treatment. Supplementary Table 5. Demographics of patients with or without anorexia [file 13023_2025_3651_MOESM2_ESM.docx]

**Supplementary Table 1. The primers used for qRT-PCR**

| Primer | Sequence |
| --- | --- |
| P1 (human *GDF15* qRT-PCR F) | GCAATGCCTGAACAGCGAC |
| P2 (human *GDF15* qRT-PCR R) | CTGAGTTCGAGTCCTCTCGG |
| P3 (human *ATF4* qRT-PCR F) | CTTGATGTCCCCCTTCGACC |
| P4 (human *ATF4* qRT-PCR R) | CTTGTCGCTGGAGAACCCAT |
| P5 (human *ATF5* qRT-PCR F) | TGGCTCCCTATGAGGTCCTTG |
| P6 (human *ATF5* qRT-PCR R) | TCAGAGAAGCCATCACCTGCC |
| P7 (human GAPDH qRT-PCR F) | GCACCGTCAAGGCTGAGAAC |
| P8 (human GAPDH qRT-PCR R) | TGGTGAAGACGCCAGTGGA |
| P9 (human CHOP qRT-PCR F) | CTTGTTCCAGCCACTCCCCAT |
| P10 (human CHOP qRT-PCR R) | TCTGAAGACAGGACCTCTTGC |
| P11 (human TRIB3 qRT-PCR F) | TGTCGCTTTGTCTTCGCTGA |
| P12 (human TRIB3 qRT-PCR R) | CCCACAGGGAATCATCTGGC |

**Supplementary Table 2 Demographics of three study groups**

|  | Late-onset MADD | MDs | Controls |
| --- | --- | --- | --- |
| Number | 41 | 23 | 46 |
| Age, years | 31.3±17.5 | 34±12.1 | 29.5±12.4 |
| %, males | 61.0%, (25) | 47.8%, (11) | 65.2%, (30) |

Note: MADD: Multiple acyl-CoA dehydrogenation deficiency; MDs: Mitochondrial diseases

**Supplementary Table 3 Clinicopathological characteristics of patients with MDs**

| Patient | gender | age | liver dysfunction | renal dysfunction | COPD | Diabetes status | CK | GDF15 |
| --- | --- | --- | --- | --- | --- | --- | --- | --- |
| 1 | F | 38 | N | N | N | N | UA | 2170.00 |
| 2 | M | 25 | N | N | N | N | 85 | 3061.00 |
| 3 | F | 23 | N | N | N | N | 102 | 4571.00 |
| 4 | M | 28 | N | N | N | N | 114 | 2185.00 |
| 5 | M | 30 | N | N | N | N | UA | 1217.00 |
| 6 | M | 24 | N | N | N | Y | UA | 1553.00 |
| 7 | M | 39 | N | N | N | N | 68 | 2384.00 |
| 8 | F | 20 | N | N | N | N | 103 | 2248.00 |
| 9 | M | 20 | N | N | N | Y | 113 | 1237.00 |
| 10 | F | 39 | N | N | N | N | 86 | 1535.00 |
| 11 | M | 36 | N | N | N | Y | 70 | 2761.00 |
| 12 | F | 56 | N | N | N | N | 107 | 3371.00 |
| 13 | M | 27 | N | N | N | N | 833 | 2811.00 |
| 14 | M | 51 | N | N | N | Y | 109 | 4039.00 |
| 15 | M | 21 | N | N | N | N | 122 | 1759.00 |
| 16 | F | 26 | N | N | N | N | 41 | 2434.00 |
| 17 | M | 45 | N | N | N | N | 262 | 2388.00 |
| 18 | M | 35 | N | N | N | N | 221 | 1658.00 |
| 19 | F | 60 | N | N | N | N | 426 | 3476.00 |
| 20 | F | 44 | N | N | N | N | 282 | 3326.00 |
| 21 | F | 32 | N | N | N | N | 76 | 1759.00 |
| 22 | F | 17 | N | N | N | N | 106 | 2536.00 |
| 23 | F | 47 | N | N | N | N | 61 | 2384.00 |

Note: MDs: Mitochondrial diseases; GDF15: Growth differentiation factor 15; M: Male; F: Female; COPD: chronic obstructive pulmonary disease; CK: creatine kinase; N: No; Y: Yes; UA: unavailable

**Supplementary Table 4 Clinical detail variations of patients before and after treatment**

| Case | sex | onset age | CK(U/L) | | urine organic acid profile | | GDF15(pg/mL) | |
| --- | --- | --- | --- | --- | --- | --- | --- | --- |
|  |  |  | before treatment | after treatment | before treatment | after treatment | before treatment | after treatment |
| 1 | M | 29 | 400-500 | UA | UA | UA | 8472.1845 | 1275.96178 |
| 2 | F | 31 | 81 | UA | glutaric aciduria | NL | 3717.67239 | 1129.2161 |
| 3 | F | 18 | 2909 | UA | glutaric aciduria | UA | 1461.00242 | 371.413141 |
| 4 | M | 22 | 259 | UA | glutaric aciduria | NL | 6188.69623 | 1162.72417 |
| 5 | F | 20 | 7856 | 4952 | glutaric aciduria | UA | 5628.4414 | 177.6325 |
| 6 | F | 51 | 342 | UA | UA | UA | 8023.92813 | 5437.86508 |
| 7 | M | 40 | 252 | 44 | UA | UA | 5623.43115 | 2299.09216 |
| 8 | F | 66 | 1188 | 26 | glutaric aciduria | NL | 7999.99445 | 1625.83958 |
| 9 | M | 36 | 433 | UA | UA | UA | 3314.30306 | 458.74 |
| 10 | M | 14 | 339 | 117 | glutaric aciduria | NL | 9030.89557 | 414.542573 |

GDF15: Growth differentiation factor 15; M: Male; F: Female; CK: creatine kinase; UA: unavailable; NL: normal

**Supplementary Table 5 Demographics of patients with or without anorexia**

|  | Patients with anorexia | Patients without anorexia |
| --- | --- | --- |
| Number | 20 | 21 |
| Age, years | 30.5±16.8 | 32.1±18.5 |
| %, males | 60.0%, (12) | 61.9%, (13) |
